# On Moving the Eyes to Flag Lucid Dreaming

**DOI:** 10.3389/fnins.2020.00361

**Published:** 2020-04-15

**Authors:** Sergio Arthuro Mota-Rolim

**Affiliations:** Brain Institute, Department of Physiology and Behavior, and Onofre Lopes University Hospital - Federal University of Rio Grande Do Norte, Natal, Brazil

**Keywords:** Lucid dream, REM (Rapid Eye Movement) sleep, scanning hypothesis, eye movement, dream imagery

## Introduction

Lucid dreaming (LD) started to be scientifically investigated through instructing dreamers to move their eyes as soon as they become lucid (Hearne, 1978; LaBerge et al., 1981). LD signaling through pre-agreed eye movements (PAEM) is possible because eye muscles are exempt from the muscular atonia that accompanies REM sleep (Aserinsky and Kleitman, 1953; Jouvet, 1962). In addition, it is hypothesized that eye movements during REM sleep relate to dreaming imagery (Roffwarg et al., 1962); however, studies that compared the direction of eye movements with dream recall yielded inconsistent results (for review see Arnulf, 2011). Moreover, it is not yet clear whether it is physiologically possible to move the eyes consciously and voluntarily during a pure REM sleep episode, as required for the PAEM. Consistently, it was found that frontal gamma activity (~40 Hz) increases during LD, suggesting that LD is a mixture of REM sleep and waking (Mota-Rolim et al., 2008; Voss et al., 2009). Besides, alpha bursts (~10 Hz) were preliminary observed during some PAEM (Mota-Rolim, 2012), which suggests that, in these cases, the PAEM may be performed in a transition from REM sleep to waking. Finally, despite being the most used technique to record LD, there is still no consensus regarding how to apply the PAEM in the lab. In this article, I will delve into the issues of recording LD through PAEM.

## The Scanning Hypothesis

The relation between eye movements during sleep and dreams was initially described by Aserinsky and Kleitman (1953), who observed that the sleeper eyes sometimes moved “rapidly, jerky, and binocularly symmetrical.” These pioneer researchers also found that upon waking up sleepers during this period—named “rapid eye movement sleep,” or “REM sleep”—most of them report dreams with intense visual imagery. Subsequently, in the first attempts to investigate the association between eye movements and dream imagery, it was observed a positive relation between the direction of rapid eye movements and gaze direction during the dream in 70–80%, as reported following awakenings (Dement and Kleitman, 1957; Dement and Wolpert, 1958; Roffwarg et al., 1962). These results gave rise to the “scanning hypothesis” (Roffwarg et al., 1962), which postulates that the eye movements during sleep are directed by the dream imagery, in a comparable way as during the waking state, in which the eyes move toward scanned objects (for comprehensive reviews, see Arnulf, 2011; and Hong et al., 2018). Based on these findings, and on the fact that during REM sleep the limb—but not eye—muscles are atonic, Hearne (1978) and LaBerge et al. (1981) developed the PAEM technique aiming to objectively record LD in the laboratory.

### Studies that Corroborate the Scanning Hypothesis

#### Physiological Conditions

After the first studies that gave rise to the scanning hypothesis (Dement and Kleitman, 1957; Dement and Wolpert, 1958; Roffwarg et al., 1962), it was found a positive relationship between gaze direction subjectively experienced during LD and the actual eye gazes objectively measured (Tholey, 1983). A subsequent study investigating non-LD found similar results (Herman et al., 1984). More recently, LaBerge et al. (2018) reported that the eye movements during tracking of a target during lucid REM sleep are similar to those of waking perception (sustained smooth pursuit) and different from those of visuomotor imagination (saccadic eye tracking). Since perceiving, imagining, and dreaming activate the same brain areas for a given sensory modality (Farah, 1988; Ishai and Sagi, 1995; O'Craven and Kanwisher, 2000; Siclari et al., 2017), LaBerge et al. (2018) argued that during dreaming (but not during imaging) there are both low competition among sensory inputs and high activation in extrastriate visual cortices. Thus, the experience of image vividness is similar to waking perception and activates the primary pursuit temporal pathway that drives the related motor regions of the cerebellum (Krauzlis, 2004). LaBerge and colleagues also found that subjective eye gazes during LD are associated with corresponding rotations of the eyes, supporting the scanning hypothesis. However, not all rapid eye movements would track the dream imagery, thus they consider that there are multiple sources of eye movements in REM sleep, and only a fraction of them scans dream images.

According to Jouvet (1967), there is a close temporal relationship between the rapid eye movements and a phasic activity that starts in the pons, then propagates to the lateral geniculate nucleus until it reaches the occipital region. These ponto-geniculo-occipital (PGO) waves were first described in cats (Jouvet et al., 1959) but exist in other mammals including macaques and baboons (Datta, 1997). Interestingly, REM sleep amount (out of total sleep time) varies considerably among terrestrial mammals: approximately 56% in the platypus, 40% in ferrets, 23% in humans, 18% in cows, and 3% in the mongoose lemur (the lowest REM sleep amount of all) (Madan and Jha, 2012). In humans, Miyauchi et al. (2009) observed an activation of the primary visual cortex associated with the rapid eye movements, which suggests the existence of PGO waves in our specie, and a link between PGO spikes, rapid eye movements and the visual aspects of dreaming.

#### Pathological Conditions

Subjects with REM sleep behavior disorder have no muscle atonia during REM sleep, and their dream reports are congruent with the abnormal behaviors (Schenck et al., 1986). When their rapid eye movements accompany a goal-oriented behavior (e.g., climbing a ladder), 90% of the cases were related to their action (Leclair-Visonneau et al., 2010), which supports the scanning hypothesis.

### Studies That Do Not Corroborate the Scanning Hypothesis

#### Physiological Conditions

After the pioneer works that compared the direction of eye movements during REM sleep with gaze direction in the dream (Dement and Kleitman, 1957; Dement and Wolpert, 1958; Roffwarg et al., 1962), two studies yielded inconsistent results, with a concordance rate varying from 9 to 32%, which was below chance (Moskowitz and Berger, 1969; Jacobs et al., 1972). In addition, some subjects awakened during phasic REM sleep (defined by rapid eye movement bursts) do not report dreaming (Siclari et al., 2013). Moreover, visual dreams are reported during both REM sleep with no rapid eye movements (tonic REM sleep) (Foulkes and Pope, 1973; Hobson et al., 2000; Hodoba et al., 2008) and non-REM sleep (Cavallero et al., 1992; Fosse et al., 2001; Mota-Rolim et al., 2015; Siclari et al., 2017). Additional studies in other animals also do not corroborate the scanning hypothesis. In monkeys, for example, Zhou and King (1997) found that some binocular rapid eye movements are not conjugated, that is, they do not move toward the same direction and thus lack a fixation point, which would prevent these eye movements to “watch” dream images.

#### Pathological Conditions

Despite the fact that congenitally blind individuals do not experience “visual” dreams and display rapid eye movements (Gross et al., 1965; Kerr et al., 1982), a recent work found that the frequency of their gazes is reduced and bears no relation with dream content (Christensen et al., 2019). In cats, when the visual cortex is removed the rapid eye movements are preserved (Jouvet, 1962), and the PGO waves, which may induce the formation of the images and other visual aspects of dreams, are generated simultaneously and in parallel with the rapid eye movements (Vanni-Mercier and Debilly, 1998).

## Lucid Dreaming as a REM Sleep to Waking Transition

In addition to the controversies surrounding the scanning hypothesis, it is unclear whether the eyes can be moved—in a voluntary and conscious way—within REM sleep, that is, without arousal or waking features. For example, the alpha rhythm power (~10 Hz) increases during LD (Ogilvie et al., 1982; Tyson et al., 1984; Mota-Rolim et al., 2008), but alpha oscillations are associated with waking state with eyes closed (Berger, 1929; Adrian and Matthews, 1934). Similarly, frontal gamma power (~40 Hz) increases during LD (Mota-Rolim et al., 2008; Voss et al., 2009), which suggests that LD is a mixture of REM sleep and waking consciousness. This supports the finding that the brain mechanisms that underlie the eye movements during sleep differ from those during wakefulness (Abe et al., 2008). In fact, frontal association areas control the eye movements during waking (together with other regions of the cingulate and parietal cortices) (Johnston and Everling, 2008), but during REM sleep these frontal areas are hypo-active (Maquet et al., 1996).

Furthermore, bursts of alpha activity during some PAEM were preliminary observed (Mota-Rolim, 2012). These alpha bursts occurring during REM sleep without muscle tone modification are classified as micro-arousals (Cantero and Atienza, 2000; Cantero et al., 2000). This suggests that, at least in some cases, the PAEM may be performed in a micro-arousal, i.e., a transitional phase from REM sleep to waking, and not within a pure REM sleep state. This may happen mainly for the naïve lucid dreamers—i.e., those who do not experience LD frequently, and who represent the vast majority of lucid dreamers. These subjects often report that they wake up as soon as they try to perform the PAEM, as if the required mental effort would induce a micro-awakening or a more superficial sleep. They also tend to wake up right after becoming lucid and have less control over the oneiric content (Mota-Rolim et al., 2013). On the other hand, Rak et al. (2015) found that narcoleptic patients—who experience fast transitions between waking and sleep—have more LD than the general population, and the mental effort needed to achieve and sustain a lucid REM sleep might be lower in these patients (Dodet et al., 2014). In a similar way, experienced lucid dreamers—a minority of people who have LD very often—have longer and more stable LD, as well as higher control over the dream content. In these people, LD may happen during a steady REM sleep state. Noteworthy, since recording LD is complex and costly, most sleep labs investigate experienced lucid dreamers, which increases the chance to successfully record an LD (but usually at the cost of small sample size). Besides, transferring these lab results to the general population (i.e., naïve lucid dreamers) should be done with caution.

## Toward a Standardization of the PAEM

The PAEM technique to flag LD during REM sleep has been widely used in physiological (LaBerge et al., 1981; Brylowski et al., 1989; Mota-Rolim et al., 2010; Dresler et al., 2012), pathological (Tang et al., 2006; Dodet et al., 2014; Oudiette et al., 2018), and artificial (Stumbrys et al., 2013; Mota-Rolim et al., 2019) conditions. Additionally, LD flagged by PAEM has also been described during non-REM sleep stages N1 (sleep onset) and N2 (superficial sleep) (LaBerge, 1980; Stumbrys and Erlacher, 2012; Mota-Rolim et al., 2015), but not during N3 (deep sleep).

Despite being the most used technique to record an LD, there is still not a consensus about how exactly PAEM should be applied, which resulted in several variations of the method, regarding mainly: (1) the number of eye movements, (2) the amount of series of eye movements, (3) the way these movements should be performed, and (4) when they should be performed. Below I detail each of these four points and suggest ways to standardize them for future studies.

1) The number of eye movements: Even though the involuntary eye movements of REM sleep being isolated, it is common to observe bursts of 2 to 5 consecutive eye movements (Arnulf, 2011). This happens especially in the elderly (Ficca et al., 1999), and resembles the PAEM. Thus, the minimum number of eye movements required to differentiate the voluntary ocular gazes from the involuntary ones that characterize REM sleep would be 6 (Mota-Rolim, 2012).
2) Amount of series of eye movements: Dreamers could also be instructed to perform more than one series of PAEM, for example, the first series when they realize that they became lucid, and another series when they believe they are close to waking up. This would improve the technique, and consequently strengthen the reliability of the study.
3) How the eye movements should be performed: While most LD researchers instruct dreamers to shift their gaze laterally (Mota-Rolim et al., 2008; Voss et al., 2009; Dresler et al., 2012), others instruct to “scan the horizon” from left to right (Dodet et al., 2014). As a way to standardize the PAEM technique, LD researchers can follow the instructions suggested by Baird et al. (2019) (adapted from LaBerge et al., 2018), which require asking the dreamer to move the eyes all the way to the left and then to the right (as if looking at each of the ears) through a continuous movement without pausing.
4) When the eye movements should be performed: In addition to during dreaming, this technique could also be practiced during the waking state (with eyes open and closed) before starting the experiment, which constitutes a valuable opportunity for researchers to view the fingerprints of each individual and also provide feedback.

## Conclusions

Despite some studies have found a relation between subjective eye movements that would scan the dream scenes and actual eyeball rotations during REM sleep, the scanning hypothesis is still controversial. More studies are also necessary to clarify whether the PAEM are realized during a pure REM sleep episode, or else mixed with (micro)-arousal/waking, especially in naïve lucid dreamers. Finally, since the PAEM constitute the most used method to scientifically study LD, a consensus on how to apply this technique in a standardized way is clearly warranted.

## Author Contributions

The author confirms being the sole contributor of this work and has approved it for publication.

### Conflict of Interest

The author declares that the research was conducted in the absence of any commercial or financial relationships that could be construed as a potential conflict of interest.
